# Structure, function, and pathology of PHF23

**DOI:** 10.3389/fcell.2025.1691318

**Published:** 2025-11-05

**Authors:** Linlin Liu, Rui Zhang, Rui Liu, Yu Liu, Yanming Ren

**Affiliations:** ^1^ Department of Neurosurgery, West China Hospital, Sichuan University, Chengdu, China; ^2^ The First College of Clinical Medicine, Chongqing Medical University, Chongqing, China; ^3^ Department of Anesthesiology, The First Affiliated Hospital, Chongqing Medical University, Chongqing, China

**Keywords:** PHF23, PHD domain, epigenetic regulation, cancer, degenerative disease

## Abstract

Plant Homeodomain Finger Protein 23 (PHF23) is a member of the Plant Homeodomain (PHD) finger protein family and has been extensively reported since its discovery. Numerous studies have demonstrated that PHF23 plays a crucial role in various biological processes, such as gene expression regulation, autophagy, and tumorigenesis. Additionally, PHF23 is associated with various diseases including various malignancies, osteoarthritis, and tuberculosis, all of which currently lack effective and targeted treatment options. Therefore, this review systematically summarizes the existing literature on PHF23, and provides a comprehensive overview of the structure and function of PHF23, and focuses on its relationship with multiple diseases. We aim to advance PHF23 research to establish it as a novel therapeutic and diagnostic target, offering new hope for patients with related diseases while reducing adverse clinical outcomes.

## 1 Introduction

The Plant Homeodomain (PHD) finger protein family is a class of proteins that contain a Zn^2+^ domain and is widely distributed in eukaryotes (Bienz, 2006; Gaurav and Kutateladze, 2023). The PHD mainly participates in epigenetic regulation and plays a crucial role in processes such as gene transcription, DNA damage repair, dynamic regulation of nucleosomes, and nuclear signal transduction (Baker et al., 2008; Black and Kutateladze, 2023; Gaurav and Kutateladze, 2023). The abnormal expression of the PHD domain can lead to the occurrence of various diseases, including cancer, immunodeficiency, and neurological disorders, etc (Musselman and Kutateladze, 2009; Mao et al., 2019; Gu et al., 2021). Given these critical functions, the PHD finger protein family represents a key target for epigenetic research and therapeutic development.

The PHD finger protein family comprises numerous members with both overlapping and distinct functions. Researchers have extensively studied some family members. For example, PHD Finger Protein 19 (PHF19) contributes to cardiac hypertrophy and multiple myeloma (Gu et al., 2021). PHD Finger Protein 5A (PHF5A), on the other hand, plays a key role in the occurrence and development of lung adenocarcinoma and glioblastoma (Mao et al., 2019). These PHD finger proteins demonstrate complex pathophysiological mechanisms in various diseases. PHD Finger Protein 23 (PHF23), first identified in 2007, represents a novel PHD family member. Studies indicate that its fusion with the nucleoporin gene NUP98 can induce acute myeloid leukemia (AML) (Reader et al., 2007).

In recent years, numerous studies have shown that PHF23 is widely expressed in various tissues and cell lines, primarily localized in the nucleus, and its functions involve multiple aspects such as cellular autophagy, tumorigenesis, and differentiation regulation (Reader et al., 2010; Wang et al., 2014; Cheng et al., 2023). Investigating PHF23’s molecular mechanisms will not only reveal its disease-related actions but also uncover new therapeutic targets. This review summarizes in detail the structure and biological functions of PHF23, with a particular focus on its relationship with various diseases.

## 2 The structure of PHF23

The PHF23 gene is located at the 17p13.1 region of human chromosome and comprises five exons (Wang et al., 2014; Katoh, 2015; Togni et al., 2015; Ning, 2016; Frigault et al., 2023). Its full-length mRNA encodes a 403-amino acid protein with a predicted molecular weight of 43.8 kDa (Wang et al., 2014). In mice, the PHF23 gene resides on chromosome 11 and encodes a 401-amino acid protein that shows high sequence similarity to human PHF23. Evolutionary analysis reveals high conservation of PHF23’s amino acid sequence across multiple species (Wang et al., 2014; Chen et al., 2021).

**FIGURE 1:**
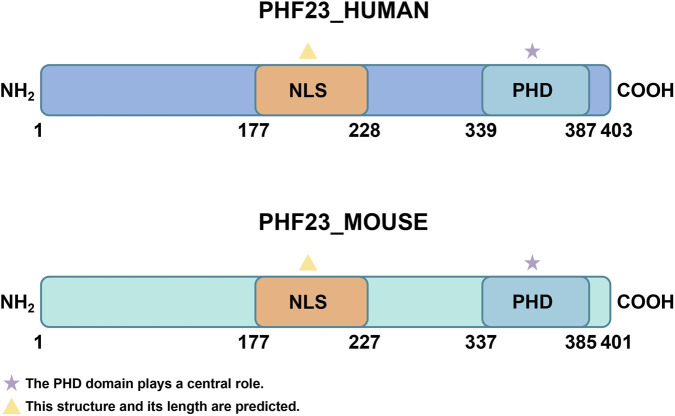
The structure of PHF23. The two most important structures of PHF23 are the PHD domain and NLS. PHD, Plant Homeodomain; NLS, Nuclear localization signal.

The PHD finger domain forms the structural core of PHF23, spanning residues 339-387 at the C-terminus (Gaurav and Kutateladze, 2023). This domain features a characteristic Cys4-His-Cys3 (C4HC3) motif that coordinates two zinc ions (Wang et al., 2014). In addition to binding to histones, the PHD finger domain can also interact with other proteins, forming a complex regulatory network (Gaurav and Kutateladze, 2023). Additionally, PHF23 is predicted to contain a nuclear localization signal (NLS) between residues 177–228 (Wang et al., 2014). This can assist it in entering the nucleus to exert its functions (Figure 1).

## 3 The functions of PHF23

### 3.1 Epigenetic regulation and cell differentiation

Histone modification represents a core epigenetic mechanism that regulates chromosome structure and gene expression, influencing numerous cellular processes (Wang et al., 2025; Zhang et al., 2025). The N-terminal tails of histones undergo various chemical modifications, mainly acetylation, phosphorylation, and methylation (Zhang et al., 2025). Histone acetylation activates gene expression by promoting chromatin relaxation (Alexandrova et al., 2025). Phosphorylation mediates the stress response and DNA damage repair, and cooperates with other modifications to regulate chromosome dynamics (Liu et al., 2025). The functional consequences of methylation depend on specific sites and degrees. H3K4me3, the trimethylation of lysine 4 on histone H3, serves as a classic example and represents a common PHD finger ligand (Lee et al., 2009; Mariani et al., 2016). PHF23’s PHD finger domain critically contributes to chromatin modification and cell differentiation through H3K4me3/2 recognition (Ning, 2016; Yu et al., 2017). Recent studies indicate that the functional cell type-specificity of PHF23 is determined by the protein complexes it recruits, along with other regulatory factors, based on its recognition of H3K4me3/2 (Wen et al., 2025). For instance, PHF23 is highly expressed in radial glial cells (RGCs) and intermediate progenitor cells (IPCs) but not in neurons (Wen et al., 2025). This expression pattern aligns with PHF23’s role in promoting the transition of RGCs and IPCs into neurons, suggesting its significant function in neurogenesis. Mechanistically, PHF23 binds to HDAC2 and inhibits its deacetylase activity toward the active histone mark H3K27ac, thereby enhancing the expression of neuronal differentiation pathway genes such as Tcf4 and Eya1 (Wen et al., 2025). Studies of the NUP98-PHF23 fusion protein reveal that the PHD finger domain maintains its native function (Zhang et al., 2020; Chen et al., 2021). This fusion protein can recognize the H3K4me3 mark through the PHD domain, thereby affecting gene expression and cell differentiation (Zhang et al., 2020; Chen et al., 2021).

### 3.2 Autophagy and apoptosis

Autophagy and apoptosis are dynamic processes that maintain cellular homeostasis and ensure cell health and function (Chen et al., 2024). Autophagy, also known as type II programmed cell death (PCD), is an important self-protective mechanism of the body (Chen et al., 2025). Through autophagy-related gene (ATG) regulation, cells selectively degrade damaged, aged, or redundant biomolecules and organelles via lysosomal digestion, generating recyclable small molecules (Chen et al., 2025; Huang et al., 2025; Ren et al., 2025). Almost all eukaryotic cells exhibit a basal level of autophagy (Chen et al., 2025). PHF23 significantly contributes to autophagy regulation (Wang et al., 2014; Frigault et al., 2023; Wang et al., 2023). LRSAM1, a RING-type E3 ubiquitin ligase, plays key roles in cellular protein quality control (PQC) and maintains global protein homeostasis, showing broad functional potential (Mishra et al., 2019; Mishra et al., 2021). Wang et al. found that the overexpression of PHF23 inhibits autophagy, while the knockout of its expression enhances autophagy (Wang et al., 2014). Mechanistic studies revealed that PHF23’s PHD finger domain mediates interaction with LRSAM1 E3 ligase, promoting LRSAM1 ubiquitination and subsequent proteasomal degradation to inhibit autophagy (Wang et al., 2014). Additionally, PHF23 affects cellular metabolism levels by regulating the AMPK and mTOR/S6K signaling pathways (Maimaitijuma et al., 2020). As a conserved eukaryotic kinase, AMPK activation upon PHF23 knockout effectively inhibits mTOR activity (Zeng et al., 2017; Saha et al., 2021). The downstream mTOR-S6K pathway, critically involved in autophagy and apoptosis regulation, induces autophagic flux when inhibited (Zeng et al., 2017; Saha et al., 2021). It is worth noting that the knockout of PHF23 has also been reported to be related to the enhancement of mitophagy, which will also lead to cell death (Maimaitijuma et al., 2020). Therefore, based on the interactions and regulation between PHF23 and the LRSAM1 E3 ligase, AMPK and mTOR/S6K signaling pathways, as well as mitophagy, PHF23 exhibits an overall negative correlation with cellular autophagy. Current studies have revealed that PHF23 is also associated with apoptosis. As a classical form of PCD, apoptosis plays a critical role in eliminating self-reactive immune cells, cancer cells, and damaged cells, thereby maintaining organismal homeostasis (Yuan and Ofengeim, 2024). The expression of PHF23 increases in human cartilage and synovium, which was induced by IL-1β through inflammatory stress (Li et al., 2019). It results in increased apoptosis level and inhibited autophagy. The precise molecular mechanism remains unclear but may involve the mTOR-S6K signaling pathway.

### 3.3 Tumorigenesis and tumor suppression

PHF23’s role in tumorigenesis has drawn significant research interest due to its dual tumor-promoting and tumor-suppressing functions (Chen et al., 2021; Cheng et al., 2023). Multiple studies demonstrate that PHF23 contributes to tumor development through diverse pathophysiological mechanisms. Through its PHD domain, PHF23 interacts with Alpha-actinin-4 (ACTN4), subsequently activating the ERK signaling pathway to stimulate tumor cell proliferation and metastasis (Cheng et al., 2023). ACTN4 belongs to the actin-binding protein family and is a non-muscle α-actinin that has long been associated with cancer development (Tentler et al., 2019). The ERK signaling cascade drives tumorigenesis by regulating critical cellular processes including proliferation, differentiation, and cell cycle control (Sugiura et al., 2021). PHF23 overexpression correlates with enhanced cell proliferation, migration, chemoresistance, and DNA repair capacity - all key factors promoting tumor formation (Cheng et al., 2023).

PHF23 demonstrates equally complex tumor-suppressive functions, primarily mediated through epigenetic regulatory mechanisms. Relevant studies have confirmed that PHF23 is identified as a 17p tumor suppressor gene (TSG), which exerts its tumor-suppressive function through its association with epigenetic regulatory mechanisms. Research indicates that, as an H3K4me3 reader, PHF23 can directly bind to the SIN3-HDAC complex and inhibit its deacetylase activity toward H3K27ac via its N-terminal domain. The formation of this PHF23-SIN3-HDAC (PSH) complex coordinates the two major active histone marks, H3K4me3 and H3K27ac, thereby further activating downstream TSGs and differentiation-related genes (Chen et al., 2021). PHF23 cooperates with chromodomain helicase DNA-binding protein (CHD) subfamily members, participating in chromatin remodeling and gene expression regulation, while displaying multifaceted roles in tumor development (Yu et al., 2017). PHF23 and CHD proteins exert their functions by binding to H3, and the types of H3 they recognize vary depending on the number of PHD finger domains in their structures (Yu et al., 2017). PHF23 contains only one PHD finger domain and recognizes H3K4me3 (Yu et al., 2017). In contrast, CHD proteins contain two PHD finger domains and lack the conserved tryptophan (trp) residues used for binding to H3K4me3, so they bind to the unmodified H3 (Yu et al., 2017). The second subfamily of CHD proteins includes CHD3, CHD4, and CHD5 (Yu et al., 2017). Notably, both CHD3 and PHF23 localize to the p53 region at 17p13.1 and function as tumor suppressors (Yu et al., 2017). Studies have found that their deletion is associated with the occurrence of cancer (Yu et al., 2017). In conclusion, the occurrence and development of tumors are a complex process, and PHF23 plays a non-negligible role in it. Elucidating these molecular pathways may reveal novel therapeutic targets for cancer treatment.

### 3.4 Other functions

**FIGURE 2:**
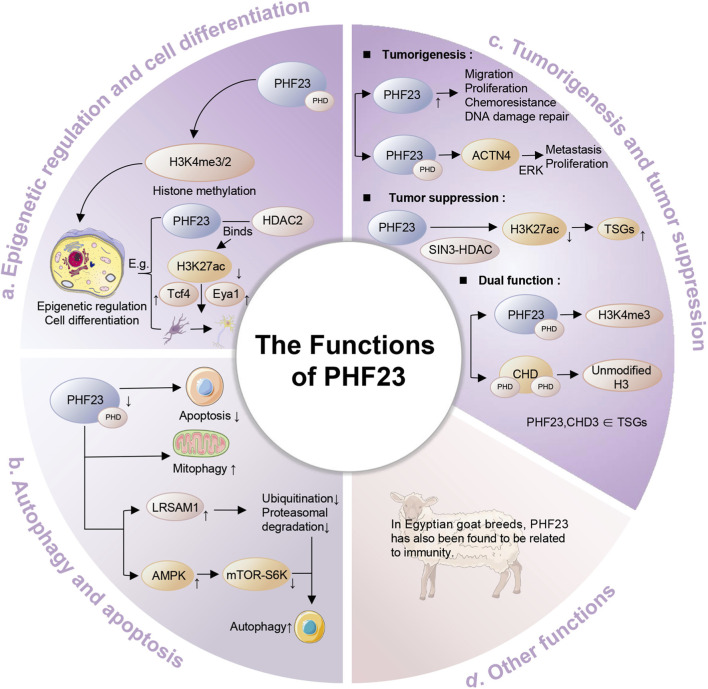
The functions of PHF23. Specifically, it includes epigenetic regulation and cell differentiation, autophagy and apoptosis, tumorigenesis and tumor suppression, immune regulation and other functions.

Studies in Egyptian goat breeds have identified PHF23’s involvement in immune regulation, though human studies require further investigation (Sallam et al., 2023). PHF23 plays an important role in multiple biological processes, and the diversity and complexity of its functions make it an important target for the study of various diseases (Figure 2).

## 4 The relationship between PHF23 and diseases

### 4.1 Cancers

#### 4.1.1 Acute myeloid leukemia

AML is a malignant clonal disease originating from bone marrow hematopoietic stem cells, characterized by the abnormal proliferation of immature myeloid cells and the blockage of differentiation (Shimony et al., 2023). AML accounts for a relatively high proportion among adult acute leukemias, and its pathogenesis involves a variety of genetic and epigenetic alterations, including gene mutations, chromosomal translocations, and abnormal epigenetic modifications (Kayser and Levis, 2023). Current AML treatment poses substantial clinical challenges, requiring multidisciplinary approaches and personalized strategies. These challenges highlight the critical need for identifying novel therapeutic targets in AML.

PHF23 plays an important role in AML. Cryptic chromosomal translocation t (11; 17) (p15; p13) generates the NUP98-PHF23 fusion protein, and this fusion protein significantly promotes oncogenesis during AML development (Reader et al., 2007; Wang et al., 2009; Gough et al., 2014; Katoh, 2015; Togni et al., 2015; Xu et al., 2016; Gough et al., 2017; Kundu et al., 2019; Hamamoto et al., 2025). NUP98-PHF23 drives leukemogenesis by aberrantly upregulating leukemia-associated genes (e.g., HOXA cluster and MEIS1), resulting in impaired hematopoietic stem cell differentiation and leukemic transformation (Gough et al., 2014; Katoh, 2015; Zhang et al., 2020; Hamamoto et al., 2025). NUP98-PHF23 recognizes the H3K4me3 mark through its PHD finger domain and maintains the characteristics of the mark, promoting the overexpression of a series of leukemia-related genes (such as the HOXA cluster and MEIS1), which in turn leads to the abnormal differentiation of hematopoietic stem cells and the occurrence of leukemia (Wang et al., 2009; Gough et al., 2014; Katoh, 2015; Gough et al., 2017; Zhang et al., 2020; Hamamoto et al., 2025). The NUP98-PHF23 fusion protein frequently occurs in AML and correlates with poor prognosis, underscoring its critical role in leukemogenesis (Ho et al., 2016; Ning, 2016). Inhibiting the histone reader function of its PHD finger domain can reverse the oncogenic effects of NUP98-PHF23, revealing potential new therapeutic targets for AML (Gough et al., 2014; Ning, 2016; Ahn et al., 2025). It is worth noting that some studies have found that the expression of PHF23 can be downregulated by enitociclib (a selective CDK9 inhibitor), which in turn allows autophagy. This provides a new and effective treatment idea for AML driven by NUP98-PHF23 (Frigault et al., 2023).

Beyond AML, PHF23 contributes to the pathogenesis of other leukemias. For example, an experiment on mice showed that the NUP98-PHF23 fusion protein, in conjunction with a Bcor gene mutation, synergistically induces pro B-1 acute lymphoblastic leukemia (ALL) (Yin et al., 2019). Among them, the NUP98-PHF23 fusion protein leads to an increase in stem cell self-renewal, and the Bcor frameshift mutation leads to impaired B-cell differentiation. These findings establish PHF23’s broad involvement in leukemogenesis and its therapeutic potential.

#### 4.1.2 Non-small cell lung cancer

Lung cancer ranks as the second most prevalent malignant tumor worldwide and exhibits the highest mortality rate (Miao et al., 2024). Among them, non-small cell lung cancer (NSCLC) is the most common pathological type of lung cancer, accounting for approximately 85% of all lung cancer cases (Miao et al., 2024). Compared with other types of lung cancer, NSCLC has significant heterogeneity, with large differences in clinical manifestations, molecular characteristics, and treatment responses, which makes its diagnosis and treatment more challenging (Miao et al., 2024).

PHF23 exerts strong oncogenic effects in NSCLC and drives tumor progression through multiple pathways. Immunofluorescence analysis reveals predominant nuclear and cytoplasmic localization of PHF23 in NSCLC cells (Cheng et al., 2023). The expression of PHF23 is positively correlated with the degree of tumor differentiation, tumor size, lymph node metastasis, and TNM staging (Cheng et al., 2023). The study by Ming Cheng et al. found that the overexpression of PHF23 significantly enhanced the proliferative ability of NSCLC cells (Cheng et al., 2023). CCK8 assays, colony formation tests, and EDU labeling consistently showed that elevated PHF23 expression enhances lung cancer cell proliferation by accelerating G1/S phase transition (Cheng et al., 2023). At the same time, it upregulated the expression of G1/S phase-related factors such as CDK4, CDK6, Cyclin D1, and Cyclin A2 (Cheng et al., 2023). In addition, PHF23 also enhanced the migratory ability of lung cancer cells by upregulating the expression of MMP2, MMP9, and N-Cadherin while downregulating the expression of E-Cadherin (Cheng et al., 2023). These results indicate that PHF23 plays a crucial role in the proliferation and processes of metastasis in NSCLC. Notably, the role of PHF23 in promoting tumor metastasis is similar to the function of heparanase in tumor invasion. Studies have found that inhibiting heparanase can suppress cell autophagy and promote apoptosis, and PHF23 also has the effects of inhibiting autophagy and promoting apoptosis (Manganelli et al., 2023). This suggests that PHF23 may have a potential connection with heparanase, thereby influencing the development of NSCLC. Ming Cheng et al. pointed out that the overexpression of PHF23 significantly increased the chemoresistance of NSCLC cells to chemotherapeutic drugs and enhanced the DNA damage repair ability, which further promoted the malignant progression of NSCLC (Cheng et al., 2023). These findings provide new potential targets for the treatment of NSCLC and lay the foundation for further research on the molecular mechanisms of PHF23 in lung cancer.

#### 4.1.3 B-cell lymphoma

B-cell lymphoma is a group of highly heterogeneous lymphoproliferative diseases, accounting for approximately 95% of all lymphomas. Diffuse large B-cell lymphoma (DLBCL) is the most common subtype (Meng et al., 2020; Liu et al., 2021). Patients typically present with lymphadenopathy and extranodal involvement, often with multi-organ system disease (Liu et al., 2021). Despite established first-line treatment protocols, many patients develop relapsed or refractory disease, creating an urgent need for novel therapeutic targets (Yamauchi and Maruyama, 2025).

In B-cell lymphoma, which is a common cancer associated with 17p deletion, PHF23 has been found to potentially suppress its development and progression (Chen et al., 2021). PHF23 is a novel 17p TSG that directly binds to the SIN3-HDAC complex through its association with epigenetic regulatory mechanisms. The resulting PSH complex ultimately activates downstream tumor suppressor genes and TSGs (Chen et al., 2021). It is noteworthy that although PHF23 haploinsufficiency is only one of hundreds of genes implicated in human cancers with 17p deletions, it is nevertheless required for these cancers’ maintenance (Chen et al., 2021). Therefore, PHF23 holds significant importance for investigating the pathophysiological mechanisms of 17p-deletion tumors (Chen et al., 2021). In addition, it has been found that the deletion of PHF23 will lead to impaired B-cell differentiation and promote the occurrence of malignancies in immature B lymphocytes.

#### 4.1.4 Breast cancer

Breast cancer (BC) is the most common malignant tumor globally and also a major cause of cancer-related deaths, especially among women (Akram et al., 2017; Barzaman et al., 2020; Katsura et al., 2022). Molecular and histological profiling classifies BC into three major subtypes: those expressing the hormone receptors of BC (estrogen receptor (ER) or progesterone receptor (PR)), those expressing human epidermal growth factor receptor 2 (HER2) of BC, and triple-negative breast cancer (TNBC) (Barzaman et al., 2020). Although treatment methods such as surgical treatment and radiotherapy are becoming less invasive and more accurate, they have a non-negligible impact on the physical and mental health of patients. In recent years, the emergence of therapies such as estrogen receptor-targeted treatment reflects a new trend in oncology: developing precise therapies directed against specific molecular and biological features of tumors (Ben-Dror et al., 2022; Neill et al., 2025). The current priority is to explore more effective targets and elucidate their underlying mechanisms.

PHF23 demonstrates potential tumor-suppressive functions in BC (Yu et al., 2017). Studies have shown that PHF23 and the CHD3 gene are both located in the 17p13.1 region, and the deletion of this region is associated with tumorigenesis. In addition, in mouse models, PHF23 and CHD3 have been identified as potential TSGs, and the deletion of these genes may promote tumor development through p53-dependent or p53-independent mechanisms (Yu et al., 2017). Therefore, further investigation into the specific molecular mechanisms by which PHF23 functions as a TSG is of significant importance for the treatment and prevention of cancer.

### 4.2 Osteoarthritis

Osteoarthritis (OA) is a common degenerative joint disease, mainly characterized by the progressive destruction of articular cartilage and the formation of osteophytes at the joint margins (Jiang, 2022). Global OA incidence continues to rise, particularly affecting elderly populations (Jiang, 2022; Minnig et al., 2024). In addition, the pathological mechanisms of OA involve multiple factors, including inflammation, abnormal cartilage metabolism, and bone remodeling (Conley et al., 2023; Courties et al., 2024). Advances in understanding OA pathophysiology are ushering in novel treatment approaches. Emerging therapies promise not only safer pain management but also potential disease modification for this painful, costly, and debilitating condition (Katz et al., 2021).

PHF23 plays an important role in the pathological process of OA by inhibiting the autophagy process and promoting the apoptosis of chondrocytes (Li et al., 2019; Maimaitijuma et al., 2020). In terms of apoptosis, some studies have shown that the expression of PHF23 increases significantly in IL-1β-stimulated chondrocytes, and its expression level is positively correlated with the apoptosis of chondrocytes (Li et al., 2019). In addition, the knockout of PHF23 can reduce the apoptosis of chondrocytes induced by IL-1β, indicating that PHF23 has the function of promoting the apoptosis of chondrocytes in OA (Li et al., 2019). In terms of autophagy, PHF23 inhibits the autophagy process of chondrocytes by regulating the AMPK and mTOR/S6K signaling pathways (Maimaitijuma et al., 2020). The study by Talatibaike Maimaitijuma et al. found that the knockout of PHF23 can significantly increase the phosphorylation level of AMPK in IL-1β-treated chondrocytes, while reducing the phosphorylation levels of mTOR and S6K, thereby enhancing autophagy activity (Maimaitijuma et al., 2020). This indicates that PHF23 exacerbates the damage of chondrocytes and the progression of OA by inhibiting autophagy (Maimaitijuma et al., 2020). In addition, PHF23 also affects the homeostasis of chondrocytes by regulating mitophagy (Sun et al., 2021). The knockout of PHF23 can increase the expression of Parkin, a marker of mitophagy, in IL-1β-treated chondrocytes, while reducing the expression of TOMM20, indicating that PHF23 further exacerbates the damage of chondrocytes by inhibiting mitophagy (Maimaitijuma et al., 2020). In addition to OA, a degenerative disease, PHF23 may also be involved in the pathological mechanism of intervertebral disc degeneration (Wang et al., 2023). Collectively, PHF23 drives degenerative pathology in OA by concurrently blocking autophagy and activating apoptosis (Li et al., 2019; Maimaitijuma et al., 2020).

### 4.3 Tuberculosis

Tuberculosis is an infectious disease caused by the *Mycobacterium tuberculosis* (*M. tuberculosis*) complex and is a major cause of death from infectious diseases globally (Furin et al., 2019; Natarajan et al., 2020). Disease progression risk correlates strongly with infection duration and host susceptibility, peaking within the first post-infection year before gradual decline (Trajman et al., 2025). Despite the availability of potentially effective antibiotics, the treatment of tuberculosis, especially drug-resistant tuberculosis, tuberculosis combined with other viral infections, etc., still faces huge challenges (Dheda et al., 2016). Unfortunately, some prominent drug targets have been found to have issues such as toxicity, insufficient *in vivo* activity, or problems with the elimination half-life (Alsayed and Gunosewoyo, 2023). It is extremely urgent to study effective targets and achieve early intervention.

**FIGURE 3:**
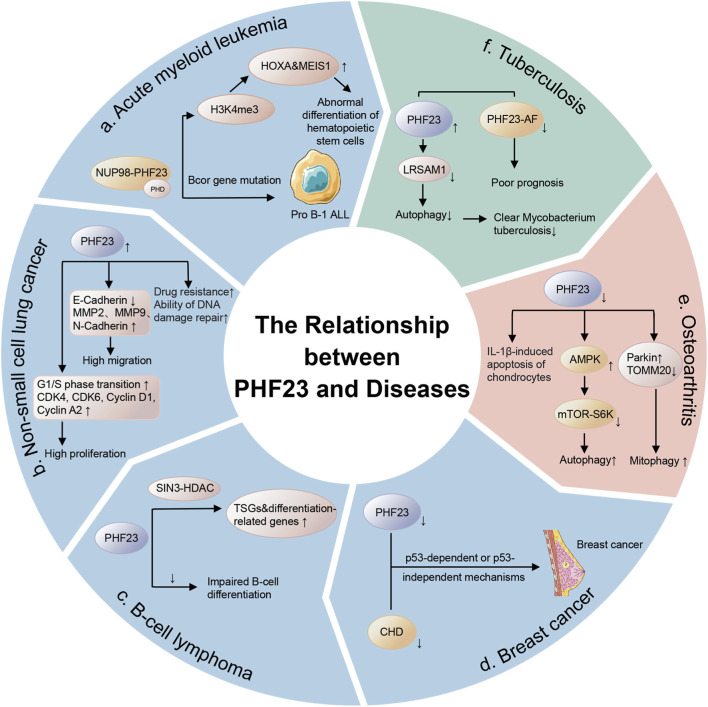
The relationship between PHF23 and diseases. These diseases include cancers such as acute myeloid leukemia, non-small cell lung cancer, B-cell lymphoma, breast cancer, and benign diseases such as osteoarthritis and tuberculosis.

PHF23 significantly correlates with tuberculosis prognosis. PHF23-AF (which replaces the first exon of the PHF23 gene) is a selective splicing event related to the prognosis of tuberculosis, and a decrease in its splicing ratio (PSI value) is associated with a poor prognosis (Lai et al., 2024). The PHF23 protein itself is a negative regulator of autophagy and functions by promoting the ubiquitination and degradation of the E3 ligase LRSAM1 (Wang et al., 2014; Lai et al., 2024). LRSAM1 has been shown to recognize a variety of bacteria and initiate the antibacterial autophagy cascade (Wang et al., 2014; Lai et al., 2024). Autophagy is an important immune mechanism for clearing *M. tuberculosis* (Lai et al., 2024). Therefore, PHF23 may hinder the clearance of *M. tuberculosis* by inhibiting autophagy, leading to an increase in bacterial load and ultimately resulting in a poor prognosis for patients with tuberculosis. The splicing variation of PHF23-AF will affect the proportion of PHF23 protein isoforms, which may thus influence its regulatory effect on autophagy and further affect the progression of tuberculosis (Lai et al., 2024) (Figure 3).

## 5 Conclusion

PHF23 is a member of the PHD finger protein family, and its research value is particularly prominent. PHF23 mainly exerts important functions through its PHD finger domain in multiple biological processes such as chromatin modification, gene expression regulation, cell differentiation, autophagy, and tumorigenesis. Its abnormal expression is associated with the occurrence and development of various diseases, including acute leukemia, NSCLC, OA, and tuberculosis. These diseases are difficult to diagnose and cure. However, the mechanisms by which PHF23 exerts its functions remain incompletely understood, particularly regarding its histone binding specificity and disease-specific pathways. Furthermore, as a novel protein, several key questions about PHF23 require clarification: whether the PHF23 gene is enriched in specific genomic regions, the mechanisms underlying its post-translational modifications, how its expression and activity are regulated, and which downstream molecular pathways it most significantly influences. These unresolved issues highlight the urgent need for further research to delineate the role of PHF23 in various diseases and to develop PHF23-targeted diagnostic and therapeutic strategies. Such advances would enable effective targeting of the molecular mechanisms driving disease pathogenesis, ultimately improving patient quality of life and clinical outcomes.
